# Hazardous work exposures and working conditions and the risk of serious injury among Latino day laborers in Houston, Texas (2014–2021)

**DOI:** 10.3389/fpubh.2025.1638490

**Published:** 2025-09-01

**Authors:** Maria Eugenia Fernandez-Esquer, Cesar Leonardo Pinzon-Gomez, Celeste Monforton, Yuan Li, Vahed Maroufy, Martha Ojeda, Anabel Rodriguez

**Affiliations:** ^1^Department of Health Promotion and Behavioral Sciences, School of Public Health, University of Texas Health Science Center, Houston, TX, United States; ^2^Department of Environmental and Occupational Health Sciences, School of Public Health, University of Texas Health Science Center, Houston, TX, United States; ^3^Department of Health and Human Performance, Texas State University, San Marcos, TX, United States; ^4^Department of Biostatistics and Data Science, School of Public Health, University of Texas Health Science Center, Houston, TX, United States; ^5^Worker Justice Alliance, Houston, TX, United States; ^6^Department of Environmental and Occupational Health, School of Public Health, Texas A&M University, College Station, TX, United States

**Keywords:** day laborer, immigrant worker, occupational safety, vulnerable workers, work environment, work-related injuries, worker safety

## Abstract

**Introduction:**

Latino day laborers (LDLs) are frequently exposed to workplace hazards that increase their risk of an injury. The purpose of this study was to assess the relative influence of worker and workplace characteristics on serious injuries reported by LDLs at four different time points. We examined the influence of demographic characteristics, hazardous chemical exposures and working conditions using data from four cross-sectional surveys conducted between 2014 and 2021.

**Methods:**

A total of 740 LDL were randomly recruited from public job hiring locations (known as “corners”) in Houston Texas to participate in surveys conducted as part of injury risk reduction studies. Spanish speaking interviewers followed a rigorously tested field methodology and administered a previously validated survey instrument. Data from four cross-sectional surveys were each examined separately and then jointly to determine the covariates associated with serious injuries.

**Results:**

Multivariable logistic regression analysis revealed significant year-to-year variability in the associations between hazardous chemical exposures, working conditions, and reported serious injuries. Gasoline exposure emerged as the strongest predictor across survey years. Analysis of the aggregated data indicates that exposure to gasoline, to dust and gasses, and to working conditions that include the risk of getting cut, and lack of ventilation, increased the odds of reporting a serious injury. By contrast, exposure to glue and adhesives was associated with a decrease in the odds of a reported serious injury. Demographic characteristics were not associated with reported injury per survey year or when data was aggregated.

**Conclusion:**

Our findings indicate that serious injury is associated with the characteristics of the workplace and not the worker, as workplace hazards were significantly associated with serious injury, while worker demographic characteristics were not. The variability in workplace hazards associated with serious injury per survey year was expected in light of the constant job rotation reported by workers. Aggregated data confirmed initial findings and also highlighted new hazards, including those associated with a decreased risk for injury. Hazards confronted by LDL need to be considered globally, as their influence may vary by job, context, circumstances, and over time. Future research should examine how different exposures interact to influence injury risk. This understanding may benefit safety training programs and guide their efforts to reduce LDL risk of injuries at work.

## Introduction

Day laborers are part of the precarious workforce in the United States (U. S.). They are hired by contractors and homeowners through informal arrangements, without the benefit of written contracts. They experience unpaid wages, unsafe work, and interpersonal mistreatment (1–6), all of which contribute to social and health disparities (7–11). Day laborers are an understudied population; the most comprehensive survey of day laborers in the U. S. was conducted more than 20 years ago, involved 2,660 respondents across 36 metropolitan areas, and found that 19% of this sample had a work-related injury in the past year that required medical attention (12).

Foreign-born Latino workers, including day laborers as a subgroup, disproportionately experience fatal traumatic injuries. They comprise 8.2% of the U. S. workforce but represented 14% of the fatalities in 2021 (13). The fatality rate for Latino workers has increased by 24% over the last decade (14). Nationally, 64% of fatal injuries among U. S. Latino workers in 2022 involved people who were foreign born. Among these deaths, 40% occurred in the construction industry (15). Certain social and economic factors contribute to higher occupational health disparities among immigrant workers, including language barriers, lack of training, discrimination, citizenship status, and fear of retaliation (12, 16, 17).

Conducting research with day laborers requires direct outreach and consistent engagement at their “corner-based” locations (e.g., street corners, parking lots, and home improvement stores). They are a mobile population, waiting to be hired, and they prioritize securing a job over any other activity. This makes it difficult to reach them to conduct research outdoors (e.g., parking lots), as participating in the surveys may interfere with their chance of being hired. Moreover, building trust and rapport with day laborers may take time, as they tend to distrust outsiders.

### Construction work is hazardous

Research indicates that Latino day laborers (LDLs) perform jobs in landscaping, moving furniture, and demolition after extreme weather events (18–23). Most jobs, however, are reported to be construction related (21–24). Although not specific to LDLs, studies of workers in the construction industry provide insight into the health and safety risks they face. Non-fatal injuries from falls, slips, and trips accounted for about one-third of Occupational Safety and Health Administration recordable incidents in the construction industry (25). Workers who suffered these injuries missed a median of 28 days of work, which illustrates the severity of their injuries (25). An analysis of 8 years of workers compensation data found that ladders were a contributing factor in 38% of non-fatal falls to a lower level (26). Many jobs performed by LDLs involve working at heights (e.g., on ladders, roofs), which is the leading cause of death among construction workers (27). In 2022, nearly 40% of fatal injuries among construction workers were due to falls, slips, and trips, and nearly all involved falls to a lower level (28).

An analysis of construction industry data from the U. S. Bureau of Labor Statistics found significantly higher fatality rates from falls among Latino workers compared to non-Hispanic White workers and among foreign-born Latino workers compared to U. S.-born Latinos (29). In a study on work-related fatalities from traumatic brain injuries (TBIs), 57% were related to falls, and 80% occurred among construction workers. The rate of TBIs was significantly higher for Latino than White workers and more profound among foreign-born Latino workers compared to U. S.-born Latinos (30). An analysis of occupational fatalities in North Carolina over a 25-year period found that the fatality rate for Latino workers was more than twice the rate for White workers. During those years, 58% of the deaths were among construction workers (31).

With respect to non-fatal injuries among construction workers, the available data by race and ethnicity are limited. An analysis of data from the Agency for Healthcare Research and Quality found that Latino construction workers were more likely to report work-related injuries as well as injuries that resulted in lost workdays (32). In one study, work-related injury inequalities among construction workers were examined using data from the National Health Interview Survey. The analysis found that, for the 14-year period ending in 2017, Latino workers reported more severe injuries than did White, non-Hispanic workers (33).

### Hazards at work experienced by day laborers

Hazards in the work environment are the most proximal determinants of an injury at work. As day laborers constantly rotate across a wide variety of jobs, they are at risk of an injury due to their exposure to a wide variety of hazards at work. Because many of their jobs are in the construction industry, the hazards include exposure to noise, extreme temperatures, dust (e.g., respirable silica, lead paint), and fumes (e.g., solder, adhesives), and electrocution, ladders, and tools (e.g., nail guns) (34). The research on the extent to which these or other hazards contribute to serious injuries among LDLs is limited. A review of the literature identified three relevant studies, discussed below.

A cross-sectional analysis of hazards reported by 21 day laborers in Chicago, Illinois, found that the most common were roofing without safety equipment, extreme temperatures, and ladders (23). A study of 183 day laborers in Seattle, Washington, found a statistically significant increased risk of exposure to noise, airborne hazards, and work at heights in day laborers who worked in construction jobs as compared to those who did not (22). A study of 217 day laborers in San Francisco, California, found that 26% experienced a work-related injury or health complaint in the previous 12 months (24).

Two reports about day laborers in the U. S. also provide insight into their working conditions. Following Hurricane Sandy in 2012, day laborers from the region were involved in clean-up activities. The most common hazards reported by the workers were unstable structures, contaminated water, hazardous waste, and mold (19). Surveys of LDLs following extreme weather events in New Orleans, Louisiana, and Houston, Texas, showed the prevalence of skin rashes, headaches, and lacerations (18, 20). These adverse health effects suggest exposure to sharp objects, fumes, and dust. Evidence also indicates that exposure to hazards varies by working context and conditions, with more hazardous work being present in disaster recovery jobs.

### Worker characteristics

Currently, there is limited information about the demographic characteristics of workers who experience serious injuries, which limits our ability to understand who is most at risk. A few studies, however, suggest that age, education, and English proficiency may contribute to the risk of an injury at work. Data from the U. S. National Health Interview Survey for construction workers (2004–2017) indicate that being a member of a racial/ethnic minority group was associated with several factors, including being of younger age and having less education. An analysis of injury rates for 2015–2017 found that the highest rates were among workers under age 25 and those between the ages of 45 and 54 years (34). Limited English proficiency also exacerbates the risk of an injury, as it can impair workers’ ability to comprehend task-related instructions and safety information (35). It should be noted, however, that the correlations with demographic factors reported in these studies were weak, indicating the need to evaluate hazards and other characteristics of construction jobs associated with injury risk (33).

### Summary and purpose of the study

The previous research indicates that LDLs are at a high risk for a serious injury at work. While research on reported injury among these workers is limited, the evidence on specific hazards and worker characteristics associated with this risk is even more limited. To assist workers in reducing their risk for injury at work, it is critical to understand how work and worker characteristics influence this risk. Furthermore, it is important to understand what hazardous chemical exposures are recurrent across different time points and which ones are associated with injury when data is aggregated regardless of the time period.

The purpose of this study is to fill this knowledge gap by exploring the association between worker (demographics) and work (hazardous chemical exposures and conditions) characteristics and injuries reported by LDLs. Our guiding research questions are: (1) is there variability in the worker and work characteristics associated with LDLs reported serious injury at specific time points? (2) Is there a set of common worker and work characteristics associated with reported serious injury regardless of time point? Our approach is unique in that it uses a rigorously tested field methodology and, for the first time, provides a systematic analysis of reported serious injuries and hazardous conditions in this vulnerable population.

## Methods

Seven unique cross-sectional surveys conducted between 2008 and 2021 were used as needs assessment tools. All except the last survey were part of a program of studies to develop and test an injury risk reduction intervention for Latino day laborers. The 2021 survey chronicled the experiences of LDL during the pandemic and included a section on working conditions. They were selected based on the relevance of their content and were initially examined for consistency and adequacy for data analysis (36–38). All surveys gathered information about demographic characteristics, work and employment characteristics, psychosocial stressors and health. Prior to survey administration, all participants were asked to provide informed consent. Study procedures were reviewed and approved by the Institutional Review Board (IRB) of the University of Texas Health Science Center at Houston Committee for the Protection of Human Subjects (36). Details of the methodology adopted across surveys have been presented elsewhere (36), and the key components are described below.

### Survey administration

We defined a “corner” as a public location (e.g., park, home improvement store, parking lot or street corner) where LDLs routinely gather and wait to be hired by contractors and homeowners. Candidate corners for each survey were observed, and their group size and location were entered into a database later used to randomly select actual interview locations. Despite variations in the main objectives of each survey, they all collected injury-related data among LDLs using the same corner selection, participant recruitment, survey measures, and administration methodology. Participants were included in surveys if they self-identified as Hispanic or Latino, they were Spanish speakers, had been looking for work at the corner for at least 3 months, and were over 18 years old. Spanish-speaking interviewers were trained extensively in survey administration at the corners, and their coding of participant responses were monitored for data quality and accuracy during the data collection period.

### Data selection and integration

**Figure 1:**
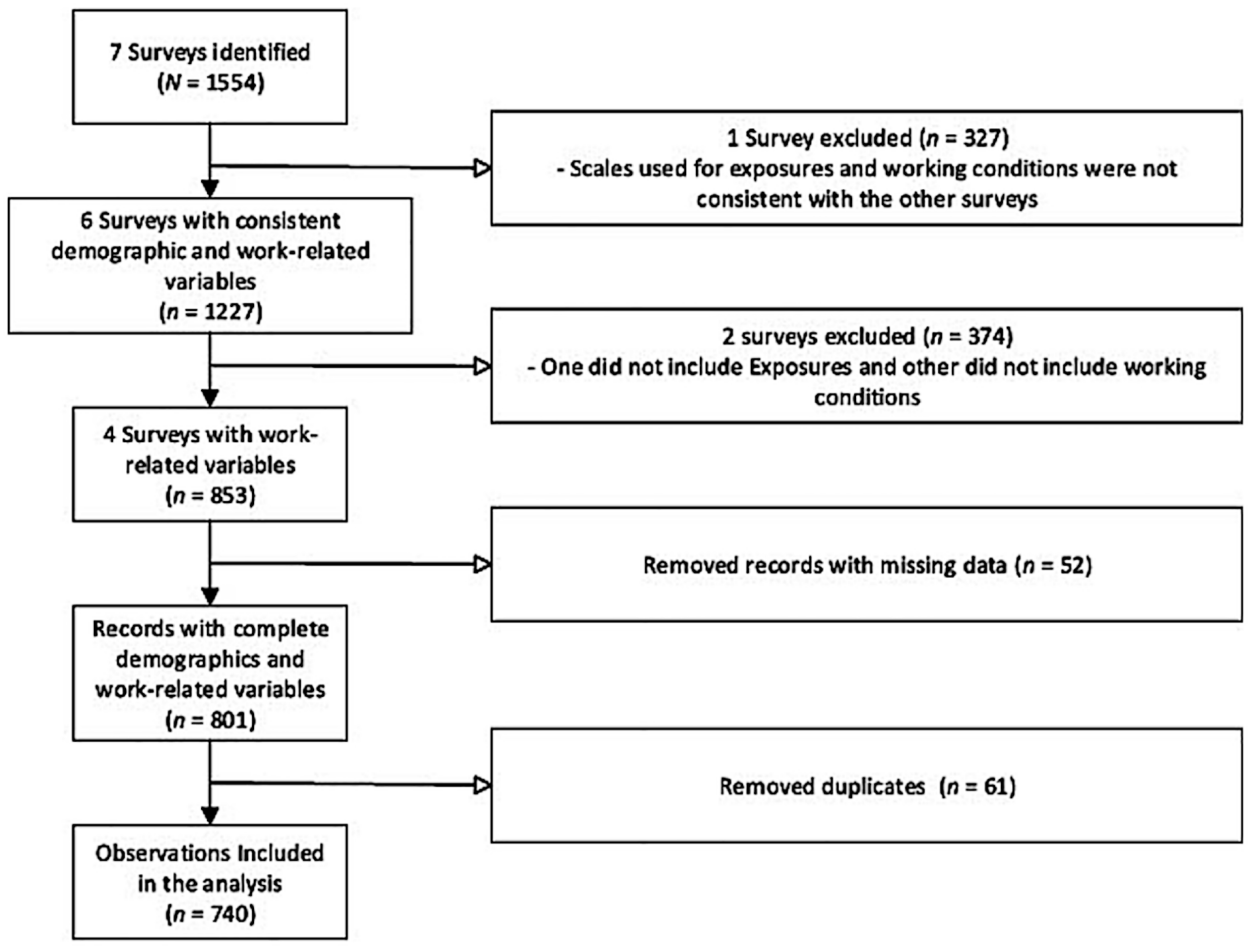
Integrated data workflow: process for merging multiple data sources into a unified analytical dataset.

To ensure comparability between surveys, we assessed consistency of question wording and response options. Of the seven available datasets, one survey was excluded because its time frame to assess exposures differed from the other surveys. A second dataset was excluded because it did not include questions assessing exposure to hazardous chemicals, and a third one was excluded because it did not include questions related to hazardous working conditions (Figure 1). In the end, we selected data from four surveys representing different time points before (2014, 2019) and during (2020, 2021) the COVID pandemic. While secular trends in the local area indicate that general working conditions may have worsened during the pandemic, our current data analysis sheds light on this assumption.

After ascertaining the comparability of data sets, we proceeded to integrate data from four selected surveys. Any records with missing values were excluded (Figure 1). Redundant records were removed and date of birth and place of origin were used to identify and eliminate duplicates. Out of 801 records, 61 (7.6%) were duplicates. We kept only the most recent record among the identified duplicates. Data was aggregated from four surveys that were conducted in 2014 (*n* = 296), 2019 (*n* = 136), 2020 (*n* = 44), and 2021 (*n* = 264). This resulting dataset contained records of 740 participants, with five demographic variables and 18 variables on hazardous chemical exposures and working conditions as described below.

### Measurement

The main outcome of interest was reported serious injury experienced in the previous year, defined as one for which the LDL missed at least 1 day of work, went to work when he was injured but thought he should have stayed home, or needed to receive medical attention from a doctor or clinic. This broad definition was chosen to facilitate comprehension and relevance among LDLs who may not be familiar with formal injury classifications. Importantly, this definition differs from OSHA’s definition of a severe injury, which includes defined outcomes such as amputation, in-patient hospitalization, or loss of an eye (39). To assess injury events, interviewers first read the definition of serious injury and then read the following question: “In the past year, have you had a serious injury related to your job as a day laborer?”

#### Demographic characteristics

Demographic variables included in the data analysis were age, place of origin, time living in the U. S. (in years), time seeking work at the corner (in years), and number of years of schooling.

#### Hazardous chemical exposures and working conditions

We defined hazardous chemical exposure and hazardous working condition as the self-reported frequency of exposure to any of the selected hazards. The hazards included in this study are based on unsafe working conditions and chemical exposures reported by LDL in previous focus groups (40, 41). Additionally, we incorporated scales in Spanish adapted for our purposes that were validated with Latino workers performing work in similar indoor and outdoor conditions (41, 42). To assess hazardous chemical exposure, the question began, “In the past 12 months, how much have you worked with …”; and for hazardous working conditions, the question began, “In the past 12 months, how frequently do your working conditions include …” Items used a four-point response scale with options 0 = never, 1 = sometimes, 2 = many times, and 3 = all the time. After the initial review of the data, due to low frequencies and practical similarity of the categories of “many times” and “all the time,” we merged the responses into the single category “many/all times.”

### Statistical analysis

To answer the first research question regarding the variability in worker and work characteristics associated with serious injury, we tested whether hazardous chemical exposures and working conditions predicted the odds of reported serious injury at three different time points. We then tested for the commonality of worker and work characteristics associated with reported serious injury across time in the integrated data set. We adopted the same statistical analysis approach to answer both research questions. First, descriptive statistics were calculated for all demographics, hazardous chemical exposures, and hazardous working conditions. Frequencies and percentages are reported for categorical variables and mean and standard deviations for continuous variables. Statistical differences in exposures and working conditions by survey year were examined using Pearson’s chi-square or Fisher’s exact test, depending on the expected frequency in each contingency table cell. Differences by injury status were tested using similar statistics when we used the integrated dataset.

We adopted a multivariable logistic regression approach to assess the extent to which worker (demographics) and work (exposure to hazardous chemicals and conditions) characteristics were associated with the odds of reporting a serious injury. Due to the limited sample size and statistical power of the 2020 survey, we excluded it from data analysis. Initially, we built univariable logistic regression models, using all 18 exposures and working conditions to select the variables and covariates significantly associated with a reported injury. Next, we used multivariable logistic regression with all the significant variables obtained from the initial univariate models. To refine the model, we applied purposeful variable selection. This method is an iterative process whereby variables are removed one at a time, starting with the variable that has the highest *p*-value. Variables were removed if their *p*-value exceeded 0.05 and if their removal did not cause a change of more than 15% in the coefficients of the remaining significant variables (43). We applied forward and backward stepwise selection to assess the final model’s robustness and the sensitivity of the model to the variable selection process (44). We computed the variance inflation factor to detect potential collinearity among the variables in our model and found it to be adequate. We deemed *p*-values below 0.05 as statistically significant. All analyses were executed in R 4.3.0.

## Results

**Figure 2:**
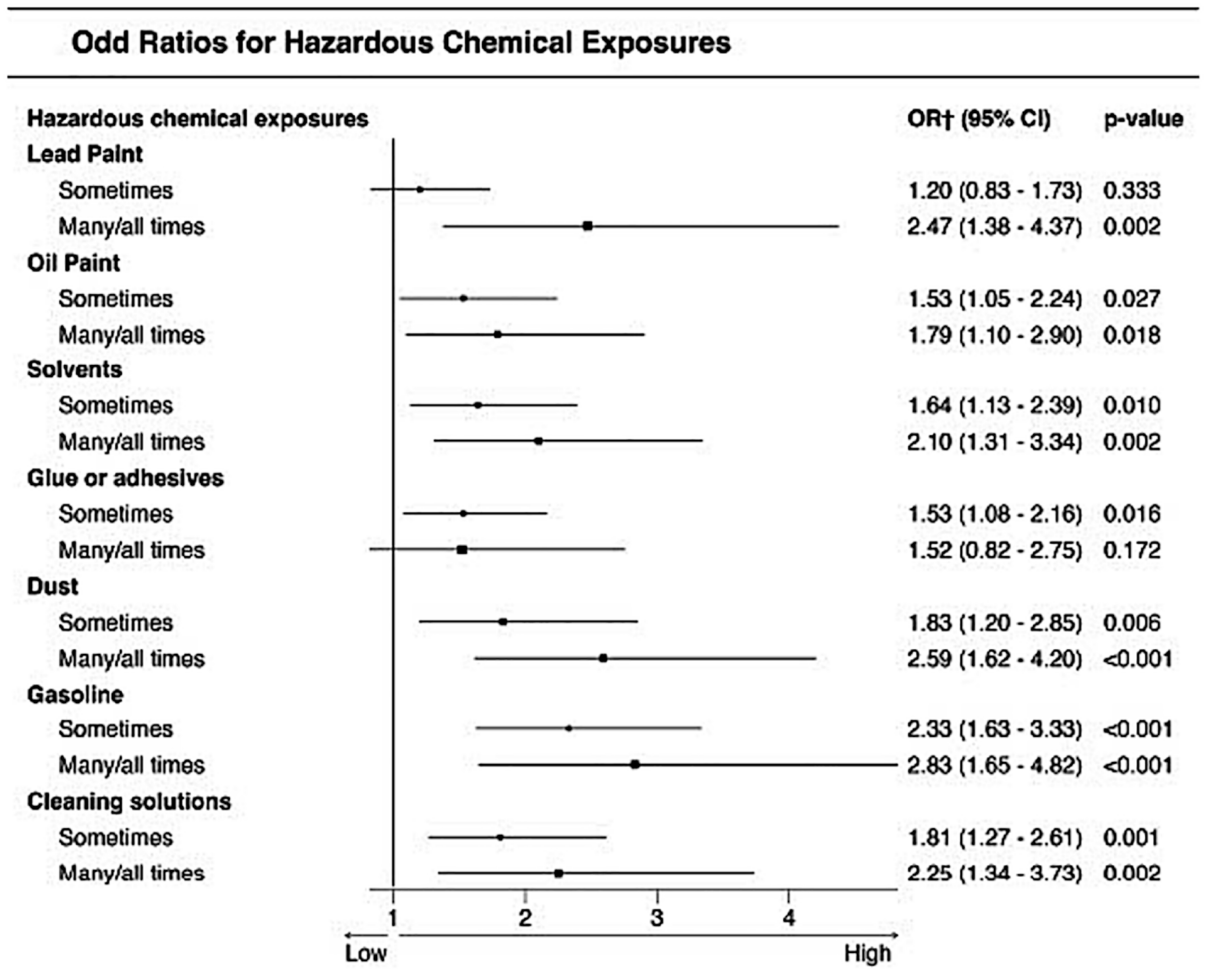
Univariate logistic regression results for hazardous chemical exposures and reported injury. ^†^Odds ratios are presented relative to the “Never” category, which was used as the reference group and is not shown in the figure.

**Figure 3:**
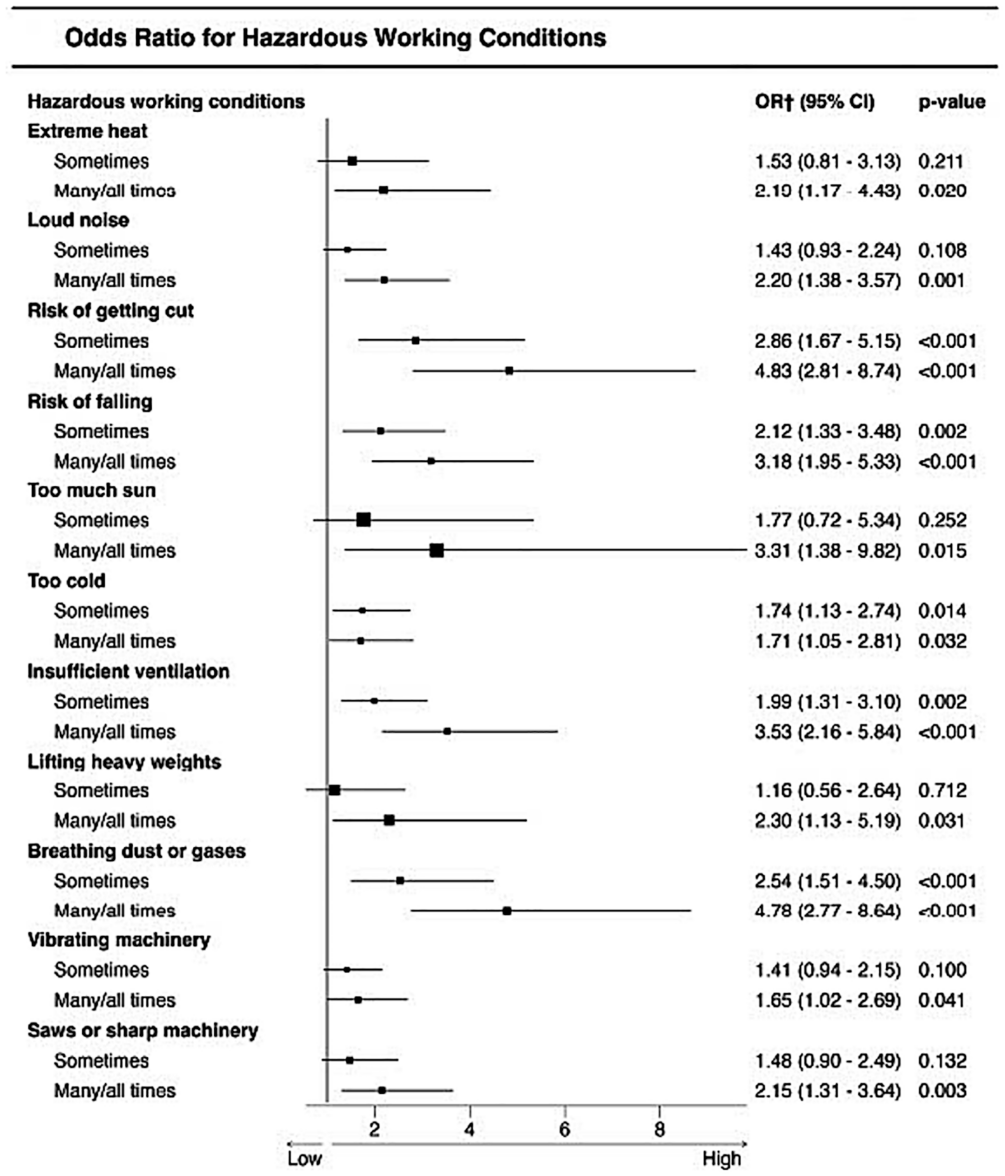
Univariate logistic regression results for working conditions and reported injury. ^†^Odds ratios are presented relative to the “Never” category, which was used as the reference group and is not shown in the figure.

We first present the results of worker and work characteristics predicting the odds of a serious injury for the first research question (variability over time) and then for the second research question (common predictors across time). For the second research question, we first present a univariate analysis for each hazardous exposure and working condition (Figures 2, 3), and then present the results of the multivariable logistic regression analysis.

### Predictors of reported serious injury by survey year

#### Descriptive statistics

The pattern of results for each survey year is presented in Table 1. We observed significant variability in the proportion of participants who reported serious injuries each year, with the lowest percentage observed in 2014 (19.6%) and the highest, in 2019 (39%). We also observed significant differences in the four surveys in all demographics (place of origin, time in the US, age, time looking for work at the corner) except years of schooling. We also found significant differences by survey year in all exposures and working conditions, except breathing dust or gasses.

**Table 1:** Demographic characteristics, hazardous chemical exposures and working conditions by survey year.

Variable	Survey year				Total (*N* = 740)	*p*-value
	2014 (*n* = 296)	2019 (*n* = 136)	2020 (*n* = 44)	2021 (*n* = 264)		
Reported serious injury						<0.001
No	238 (80.4%)	83 (61.0%)	34 (77.3%)	190 (72.0%)	545 (73.6%)	
Yes	59 (19.6%)	53 (39.0%)	10 (22.7%)	74 (28.0%)	195 (26.4%)	
Demographics						
Place of origin						0.002
Cuba	18 (6.1%)	9 (6.6%)	3 (6.8%)	38 (14.4%)	68 (9.2%)	
El Salvador	45 (15.2%)	15 (11.0%)	10 (22.7%)	25 (9.5%)	95 (12.8%)	
Honduras	62 (20.9%)	31 (22.8%)	6 (13.6%)	56 (21.2%)	155 (20.9%)	
Mexico	128 (43.2%)	48 (35.3%)	15 (34.1%)	108 (40.9%)	299 (40.4%)	
Other (Puerto Rico, Nicaragua, Guatemala)	43 (14.5%)	33 (24.3%)	10 (22.7%)	37 (14.0%)	123 (16.6%)	
Time in the US (years)						<0.001
Less than 1 year	33 (11.1%)	11 (8.1%)	0(0.0%)	25 (9.5%)	69 (9.3%)	
1 to 4 years	39 (13.2%)	28 (20.6%)	10 (22.7%)	58 (22.0%)	135 (18.2%)	
5 to 9 years	75 (25.3%)	32 (23.5%)	7 (15.9%)	30 (11.4%)	144 (19.5%)	
10 years or more	149 (50.3%)	65 (47.8%)	27 (61.4%)	151 (57.2%)	392.(53.0%)	
Age						0.010
Less than 30 years	18 (6.1%)	19 (14.0%)	4 (9.1%)	29 (11.0%)	70 (9.5%)	
30 to 39 years	88 (29.7%)	27 (19.9%)	7 (15.9%)	52 (19.7%)	174 (23.5%)	
40 to 49 years	111 (37.5%)	42 (30.9%)	17 (38.6%)	88 (33.3%)	258 (34.9%)	
50 years or more	79 (26.7%)	48 (35.3%)	16 (36.4%)	95 (36.0%)	238 (32.2%)	
Time at the corner (years)						0.014
Less than 6 months	75 (25.3%)	19 (14.0%)	9 (20.5%)	55 (20.8%)	158 (21.4%)	
6 to 11 months	50 (16.9%)	10 (7.4%)	6 (13.6%)	28 (10.6%)	94 (12.7%)	
1 to 4 years	98 (33.1%)	60 (44.1%)	16 (36.4%)	106 (40.2%)	280 (37.8%)	
5 years or more	73 (24.7%)	47 (34.6%)	13 (29.5%)	75 (28.4%)	208 (28.1%)	
Years of schooling						0.309
Less than 3 years	48 (16.2%)	30 (22.1%)	5 (11.4%)	54 (20.5%)	137 (18.5%)	
3 to 6 years	95 (32.1%)	39 (28.7%)	11 (25.0%)	68 (25.8%)	213 (28.8%)	
7 years or more	153 (51.7%)	67 (49.3%)	28 (63.6%)	142 (53.8%)	390 (52.7%)	
Hazardous chemical exposures						
Lead paint						<0.001
Never	139 (47.0%)	96 (70.6%)	36 (81.8%)	202 (76.5%)	473 (63.9%)	
Sometimes	132 (44.6%)	26 (19.1%)	7 (15.9%)	47 (17.8%)	212 (28.6%)	
Many/all times	25 (8.4%)	14 (10.3%)	1 (2.3%)	15 (5.7%)	55 (7.4%)	
Oil Paint						<0.001
Never	77 (26.0%)	45 (33.1%)	24 (54.5%)	119 (45.1%)	265 (35.8%)	
Sometimes	160 (54.1%)	69 (50.7%)	15 (34.1%)	109 (41.3%)	353 (47.7%)	
Many/all times	59 (19.9%)	22 (16.2%)	5 (11.4%)	36 (13.6%)	122 (16.5%)	
Solvents						<0.001
Never	87 (29.4%)	43 (31.6%)	28 (63.6%)	137 (51.9%)	295 (39.9%)	
Sometimes	155 (52.4%)	71 (52.2%)	8 (18.2%)	86 (32.6%)	320 (43.2%)	
Many/all times	54 (18.2%)	22 (16.2%)	8 (18.2%)	41 (15.5%)	125 (16.9%)	
Glue or adhesives						<0.001
Never	109 (36.8%)	68 (50.0%)	29 (65.9%)	174 (65.9%)	380 (51.4%)	
Sometimes	159 (53.7%)	55 (40.4%)	14 (31.8%)	73 (27.7%)	301 (40.7%)	
Many/all times	28 (9.5%)	13 (9.6%)	1 (2.3%)	17 (6.4%)	59 (8.0%)	
Dust						<0.001
Never	60 (20.3%)	43 (31.6%)	20 (45.5%)	82 (31.1%)	205 (27.7%)	
Sometimes	166 (56.1%)	69 (50.7%)	17 (38.6%)	102 (38.6%)	354 (47.8%)	
Many/all times	70 (23.6%)	24 (17.6%)	7 (15.9%)	80 (30.3%)	181 (24.5%)	
Gasoline						<0.001
Never	152 (51.4%)	69 (50.7%)	28 (63.6%)	181 (68.6%)	430 (58.1%)	
Sometimes	115 (38.9%)	54 (39.7%)	15 (34.1%)	56 (21.2%)	240 (32.4%)	
Many/all times	29 (9.8%)	13 (9.6%)	1 (2.3%)	27 (10.2%)	70 (9.5%)	
Cleaning solutions						<0.001
Never	121 (40.9%)	41 (30.1%)	26 (59.1%)	147 (55.7%)	335 (45.3%)	
Sometimes	141 (47.6%)	75 (55.1%)	16 (36.4%)	83 (31.4%)	315 (42.6%)	
Many/all times	34 (11.5%)	20 (14.7%)	2 (4.5%)	34 (12.9%)	90 (12.2%)	
Hazardous working conditions						
Extreme heat						<0.001
Never	32 (10.8%)	8 (5.9%)	6 (13.6%)	25 (9.5%)	71 (9.6%)	
Sometimes	169 (57.1%)	61 (44.9%)	12 (27.3%)	86 (32.6%)	328 (44.3%)	
Many/all times	95 (32.1%)	67 (49.3%)	26 (59.1%)	153 (58.0%)	341 (46.1%)	
Loud noise						0.007
Never	69 (23.3%)	22 (16.2%)	17 (38.6%)	73 (27.7%)	181 (24.5%)	
Sometimes	161 (54.4%)	70 (51.5%)	20 (45.5%)	117 (44.3%)	368 (49.7%)	
Many/all times	66 (22.3%)	44 (32.4%)	7 (15.9%)	74 (28.0%)	191 (25.8%)	
Risk of getting cut						0.003
Never	49 (16.6%)	19 (14.0%)	11 (25.0%)	78 (29.5%)	157 (21.2%)	
Sometimes	141 (47.6%)	70 (51.5%)	17 (38.6%)	106 (40.2%)	334 (45.1%)	
Many/all times	106 (35.8%)	47 (34.6%)	16 (36.4%)	80 (30.3%)	249 (33.6%)	
Risk of falling						<0.001
Never	54 (18.2%)	22 (16.2%)	13 (29.5%)	88 (33.3%)	177 (23.9%)	
Sometimes	154 (52.0%)	66 (48.5%)	20 (45.5%)	111 (42.0%)	351 (47.4%)	
Many/all times	88 (29.7%)	48 (35.3%)	11 (25.0%)	65 (24.6%)	212 (28.6%)	
Too much sun						0.002
Never	19 (6.4%)	6 (4.4%)	5 (11.4%)	10 (3.8%)	40 (5.4%)	
Sometimes	139 (47.0%)	42 (30.9%)	17 (38.6%)	94 (35.6%)	292 (39.5%)	
Many/all times	138 (46.6%)	88 (64.7%)	22 (50.0%)	160 (60.6%)	408 (55.1%)	
Too cold						<0.001
Never	33 (11.1%)	10 (7.4%)	12 (27.3%)	120 (45.5%)	175 (23.6%)	
Sometimes	167 (56.4%)	71 (52.2%)	28 (63.6%)	102 (38.6%)	368 (49.7%)	
Many/all times	96 (32.4%)	55 (40.4%)	4 (9.1%)	42 (15.9%)	197 (26.6%)	
Insufficient ventilation						<0.001
Never	68 (23.0%)	29 (21.3%)	25 (56.8%)	93 (35.2%)	215 (29.1%)	
Sometimes	177 (59.8%)	81 (59.6%)	16 (36.4%)	108 (40.9%)	382 (51.6%)	
Many/all times	51 (17.2%)	26 (19.1%)	3 (6.8%)	63 (23.9%)	143 (19.3%)	
Lifting heavy weights						0.045
Never	16 (5.4%)	8 (5.9%)	5 (11.4%)	21 (8.0%)	50 (6.8%)	
Sometimes	143 (48.3%)	48 (35.3%)	23 (52.3%)	127 (48.1%)	341 (46.1%)	
Many all times	137 (46.3%)	80 (58.8%)	16 (36.4%)	116 (43.9%)	349 (47.2%)	
Breathing dust or gasses						0.072
Never	55 (18.6%)	20 (14.7%)	15 (34.1%)	63 (23.9%)	153 (20.7%)	
Sometimes	161 (54.4%)	76 (55.9%)	18 (40.9%)	124 (47.0%)	379 (51.2%)	
Many/all times	80 (27.0%)	40 (29.4%)	11 (25.0%)	77 (29.2%)	208 (28.1%)	
Vibrating machinery						<0.001
Never	58 (19.6%)	25 (18.4%)	21 (47.7%)	91 (34.5%)	195 (26.4%)	
Sometimes	187 (63.2%)	69 (50.7%)	17 (38.6%)	115 (43.6%)	388 (52.4%)	
Many/all times	51 (17.2%)	42 (30.9%)	6 (13.6%)	58 (22.0%)	157 (21.2%)	
Saws or sharp machinery						<0.001
Never	35 (11.8%)	16 (11.8%)	8 (18.2%)	73 (27.7%)	132 (17.8%)	
Sometimes	148 (50.0%)	64 (47.1%)	16 (36.4%)	108 (40.9%)	336 (45.4%)	
Many/all times	113 (38.2%)	56 (41.2%)	20 (45.5%)	83 (31.4%)	272 (36.8%)	

#### Multivariable analysis

The logistic regression results by survey year are presented in Table 2. The main findings for each survey are presented below.

**Table 2:** Final model results of the multivariable logistic regressions by survey year.

Variable	Estimate	*SE*	Statistic	*p*-value	OR	95% CI		
2014 Survey								
Gasoline								
Never (ref)	–	–	–	–	–		–	
Sometimes	0.941	0.327	2.878	0.004	2.563	1.361–4.933		
Many/all times	0.654	0.519	1.261	0.207	1.924	0.658–5.153		
Lead paint								
Never (ref)	–	–	–	–	–		–	
Sometimes	0.534	0.325	1.640	0.101	1.705	0.906–3.263		
Many/all times	0.995	0.498	1.997	0.046	2.706	0.993–7.133		
2019 Survey								
Place of origin								
Mexico (ref)	–	–	–	–	–		–	
Cuba	−0.375	1.104	−0.340	0.734	0.687	0.059–5.196		
El Salvador	0.390	0.949	0.411	0.681	1.477	0.227–9.779		
Honduras	1.136	0.735	1.546	0.122	3.114	0.762–14.049		
Other (Nicaragua, Puerto Rico, Guatemala)	0.927	0.680	1.363	0.173	2.526	0.676–9.966		
Time in the US								
Less than 1 year (ref)	–	–	–	–	–		–	
1 to 4 years	−0.816	1.024	−0.797	0.426	0.442	0.058–3.439		
5 to 9 years	0.024	0.963	0.025	0.980	1.024	0.154–7.215		
10 years or more	0.689	0.923	0.747	0.455	1.992	0.328–13.202		
Years of schooling								
Less than 3 years (ref)								
3 to 6 years	0.287	0.678	0.423	0.673	1.332	0.349–5.115		
7 years or more	−0.585	0.762	−0.768	0.443	0.557	0.121–2.468		
Lead Paint								
Never (ref)	–	–	–	–	–		–	
Sometimes	0.271	0.617	0.439	0.660	1.311	0.384–4.424		
Many/all times	1.809	0.809	2.237	0.025	6.107	1.306–32.879		
Glue/adhesives								
Never (ref)	–	–	–	–	–		–	
Sometimes	1.194	0.610	1.957	0.050	3.301	1.010–11.306		
Many/all times	−0.784	1.130	−0.694	0.488	0.457	0.039–3.570		
Gasoline								
Never (ref)	–	–	–	–	–		–	
Sometimes	0.522	0.609	0.858	0.391	1.686	0.506–5.650		
Many/all times	1.315	1.114	1.180	0.238	3.724	0.424–34.757		
Risk of getting cut								
Never (ref)	–	–	–	–	–		–	
Sometimes	1.146	0.879	1.304	0.192	3.145	0.601–19.921		
Many/All times	0.202	0.978	0.207	0.836	1.224	0.187–9.161		
Too cold								
Never (ref)	–	–	–	–	–		–	
Sometimes	0.043	1.030	0.042	0.967	1.044	0.154–10.078		
Many/all times	−1.349	1.126	−1.198	0.231	0.259	0.030–2.831		
Insufficient ventilation								
Never (ref)	–	–	–	–	–		–	
Sometimes	0.640	0.717	0.892	0.372	1.897	0.482–8.382		
Many/all times	2.007	0.974	2.060	0.039	7.442	1.169–55.651		
2021 Survey								
Years of schooling								
Less than 3 years (ref)								
3 to 6 years	−0.527	0.451	−1.167	0.243	0.591	0.241–1.427		
7 years or more	−0.828	0.408	−2.028	0.043	0.437	0.195–0.973		
Lead paint								
Never (ref)	–	–	–	–	–		–	
Sometimes	−1.212	0.447	−2.715	0.007	0.298	0.120–0.695		
Many/all times	−0.945	0.716	−1.319	0.187	0.389	0.088–1.509		
Solvents								
Never (ref)	–	–	–	–	–		–	
Sometimes	1.118	0.366	3.054	0.002	3.058	1.504–6.351		
Many/all times	1.397	0.496	2.819	0.005	4.045	1.537–10.853		
Gasoline								
Never (ref)	–	–	–	–	–		–	
Sometimes	0.726	0.370	1.963	0.050	2.066	0.997–4.270		
Many/all times	0.987	0.509	1.942	0.052	2.684	0.983–7.321		
Risk of getting cut								
Never (ref)	–	–	–	–	–		–	
Sometimes	0.414	0.440	0.941	0.347	1.512	0.649–3.681		
Many/all times	1.068	0.459	2.325	0.020	2.910	1.201–7.355		
Too cold								
Never (ref)	–	–	–	–	–		–	
Sometimes	0.528	0.355	1.487	0.137	1.695	0.848–3.426		
Many/all times	0.650	0.459	1.415	0.157	1.916	0.770–4.708		
Too hot								
Never (ref)	–	–	–	–	–		–	
Sometimes	0.822	0.487	1.688	0.091	2.275	0.908–6.230		
Many/all times	1.060	0.515	2.059	0.040	2.887	1.081–8.275		

Results of the 2014 survey indicate that the odds of a serious injury were 2.56 times higher for workers exposed “sometimes” to gasoline (95% CI: 1.36–4.93, *p* = 0.004). Frequent lead paint exposure (“many/all times”) had 2.71 odds of a serious injury compared to workers never exposed (95% CI: 0.99–7.13, *p* = 0.046).

The 2019 survey indicates that the odds of a serious injury were significantly higher (6.11 times) for workers frequently exposed (“many/all times”) to lead paint (95% CI: 1.31–32.88, *p* = 0.025) and to insufficient ventilation (7.44 times) compared to workers never exposed (95% CI: 1.17–55.65, *p* = 0.039). Occasional exposure to glue/adhesives (“sometimes”) also influenced the odds of injury, with an odds ratio of 3.30 (95% CI: 1.01–11.31, *p* = 0.050).

The 2021 survey results presented in Table 2 indicate that the odds of reporting a serious injury were significantly associated with multiple factors that increase or decrease its occurrence. Workers with more schooling (i.e., 7 or more years) had 56% lower odds of a serious injury compared to those with less than 3 years of schooling (OR = 0.44, 95% CI: 0.20–0.97, *p* = 0.043). Exposure to lead paint “sometimes” was associated with 70% lower odds of an injury compared to those never exposed (OR = 0.30, 95% CI: 0.12–0.70, *p* = 0.007). Exposure to solvents, by contrast, significantly increased the odds of an injury. Workers exposed “many/all times” had odds of a serious injury that were 4.05 times higher (95% CI: 1.54–10.85, *p* = 0.005), and those exposed “sometimes” had odds that were 3.06 times higher (95% CI: 1.50–6.35, *p* = 0.002) than those never exposed. Participants reporting exposure to gasoline “many/all times” had marginally higher odds (OR = 2.68, 95% CI: 0.98–7.32, *p* = 0.052) of reporting a serious injury, while those that reported being exposed “sometimes” that has a similar trend (OR = 2.07, 95% CI: 1.00–4.27, *p* = 0.050). Workers exposed to the risk of getting cut “many/all times” had 2.91 higher odds of reporting a serious injury compared to those never exposed (95% CI: 1.20–7.36, *p* = 0.020). Finally, participants who reported breathing dust or gasses “many/all times” had significantly increased odds of reporting a serious injury (OR = 2.98, 95% CI: 1.08–8.28, *p* = 0.040).

To summarize, we found that out of 23 worker and work characteristics, 21 significantly changed over time. We then tested their influence in a multivariable logistic regression analysis and found that only lead paint and gasoline predicted the odds of reported serious injury. Exposure to lead paint increased the odds of serious injury by 2.7 times in 2014, and by 6.1 times in 2019, but in 2021 it decreased the odds of injury to 0.39 times. Exposure to gasoline also emerged as a significant predictor in two of the three surveys. Compared to never being exposed, participants who reported occasional exposure to gasoline in 2014 and 2021 were 2.6 times and 2.1 times more likely to report a serious injury, respectively.

### Predictors of reported injury over time

#### Descriptive statistics

Demographic characteristics of participants in the integrated dataset are presented in Table 3. In brief, the majority of LDLs were from Mexico (40.4%) and Honduras (20.9%). Other places of origin for study participants were El Salvador (12.8%), Cuba (9.2%), and Guatemala, Nicaragua, and Puerto Rico (16.6% combined). On average, study participants were 44.4 years of age and reported 7.6 years of schooling. Mean time in the U. S. and mean time seeking work at the corners were 13.9 and 4.5 years, respectively. Chi-square test results indicate that there were no significant differences in demographic characteristics between those who reported a serious injury in the past 12 months and those who did not. Specifically, the distribution of participants’ place of origin, time in the U. S., age, time seeking employment at corners, and years of schooling did not differ significantly by reported serious injury status.

**Table 3:** Demographic characteristics, hazardous chemical exposures and working conditions by reported serious injury status in the integrated dataset.

Variable	Serious injury in the past year		Total (*N* = 740)	*p*-value
	No	Yes		
Serious injury	545 (73.7%)	195 (26.3%)	740 (100%)	
Demographics				
Place of origin				0.755
Cuba	51 (9.4%)	17 (8.7%)	68 (9.2%)	
El Salvador	72 (13.2%)	23 (11.8%)	95 (12.8%)	
Honduras	111 (20.4%)	44 (22.6%)	155 (20.9%)	
Mexico	225 (41.3%)	74 (37.9%)	299 (40.4%)	
Other (Nicaragua, Puerto Rico, Guatemala)	86 (15.8%)	37 (19.0%)	123 (16.6%)	
Time in the US (mean = 13.9, SD = 11.2)				0.679
Less than 1 year	53 (9.7%)	16 (8.2%)	69 (9.3%)	
1 to 4 years	100 (18.3%)	35 (17.9%)	135 (18.2%)	
5 to 9 years	110 (20.2%)	34 (17.4%)	144 (19.5%)	
10 years or more	282 (51.7%)	110 (56.4%)	392 (53.0%)	
Age (mean = 44.4, SD = 11.3)				0.547
Less than 30 years	49 (9.0%)	21 (10.8%)	70 (9.5%)	
30 to 39 years	132 (24.2%)	42 (21.5%)	174 (23.5%)	
40 to 49 years	184 (33.8%)	74 (37.9%)	258 (34.9%)	
50 years or more	180 (33.0%)	58 (29.7%)	238 (32.2%)	
Time at the corner (mean = 4.5, SD = 5.6)				0.505
Less than 6 months	121 (22.2%)	37 (19.0%)	158 (21.4%)	
6 months to 11 months	73 (13.4%)	21 (10.8%)	94 (12.7%)	
1 year to 4 years	203 (37.2%)	77 (39.5%)	280 (37.8%)	
5 years or more	148 (27.2%)	60 (30.8%)	208 (28.1%)	
Years of schooling (mean = 7.6, SD = 4.0)				0.264
Less than 3 years	97 (17.8%)	40 (20.5%)	137 (18.5%)	
3 to 6 years	151 (27.7%)	62 (31.8%)	213 (28.8%)	
7 years or more	297 (54.5%)	93 (47.7%)	390 (52.7%)	
Hazardous chemical exposures				
Lead paint				0.007
Never	360 (66.1%)	113 (57.9%)	473 (63.9%)	
Sometimes	154 (28.3%)	58 (29.7%)	212 (28.6%)	
Many/all times	31 (5.7%)	24 (12.3%)	55 (7.4%)	
Oil paint				0.027
Never	210 (38.5%)	55 (28.2%)	265 (35.8%)	
Sometimes	252 (46.2%)	101 (51.8%)	353 (47.7%)	
Many/all times	83 (15.2%)	39 (20.0%)	122 (16.5%)	
Solvents				0.003
Never	236 (43.3%)	59 (30.3%)	295 (39.9%)	
Sometimes	227 (41.7%)	93 (47.7%)	320 (43.2%)	
Many/all times	82 (15.0%)	43 (22.1%)	125 (16.9%)	
Glue or adhesives				0.041
Never	295 (54.1%)	85 (43.6%)	380 (51.4%)	
Sometimes	209 (38.3%)	92 (47.2%)	301 (40.7%)	
Many/all times	41 (7.5%)	18 (9.2%)	59 (8.0%)	
Dust				<0.001
Never	170 (31.2%)	35 (17.9%)	205 (27.7%)	
Sometimes	257 (47.2%)	97 (49.7%)	354 (47.8%)	
Many/all times	118 (21.7%)	63 (32.3%)	181 (24.5%)	
Gasoline				<0.001
Never	348 (63.9%)	82 (42.1%)	430 (58.1%)	
Sometimes	155 (28.4%)	85 (43.6%)	240 (32.4%)	
Many/all times	42 (7.7%)	28 (14.4%)	70 (9.5%)	
Cleaning solutions				<0.001
Never	269 (49.4%)	66 (33.8%)	335 (45.3%)	
Sometimes	218 (40.0%)	97 (49.7%)	315 (42.6%)	
Many/all times	58 (10.6%)	32 (16.4%)	90 (12.2%)	
Hazardous working conditions				
Extreme heat				0.02
Never	59 (10.8%)	12 (6.2%)	71 (9.6%)	
Sometimes	250 (45.9%)	78 (40.0%)	328 (44.3%)	
Many/all times	236 (43.3%)	105 (53.8%)	341 (46.1%)	
Loud noise				0.003
Never	146 (26.8%)	35 (17.9%)	181 (24.5%)	
Sometimes	274 (50.3%)	94 (48.2%)	368 (49.7%)	
Many/all times	125 (22.9%)	66 (33.8%)	191 (25.8%)	
Risk of getting cut				<0.001
Never	140 (25.7%)	17 (8.7%)	157 (21.2%)	
Sometimes	248 (45.5%)	86 (44.1%)	334 (45.1%)	
Many/all times	157 (28.8%)	92 (47.2%)	249 (33.6%)	
Risk of falling				<0.001
Never	151 (27.7%)	26 (13.3%)	177 (23.9%)	
Sometimes	257 (47.2%)	94 (48.2%)	351 (47.4%)	
Many/all times	137 (25.1%)	75 (38.5%)	212 (28.6%)	
Too much sun				<0.001
Never	35 (6.4%)	5 (2.6%)	40 (5.4%)	
Sometimes	233 (42.8%)	59 (30.3%)	292 (39.5%)	
Many/all times	277 (50.8%)	131 (67.2%)	408 (55.1%)	
Too cold				0.036
Never	142 (26.1%)	33 (16.9%)	175 (23.6%)	
Sometimes	262 (48.1%)	106 (54.4%)	368 (49.7%)	
Many/all times	141 (25.9%)	56 (28.7%)	197 (26.6%)	
Insufficient ventilation				<0.001
Never	181 (33.2%)	34 (17.4%)	215 (29.1%)	
Sometimes	278 (51.0%)	104 (53.3%)	382 (51.6%)	
Many/all times	86 (15.8%)	57 (29.2%)	143 (19.3%)	
Lifting heavy weights				<0.001
Never	41 (7.5%)	9 (4.6%)	50 (6.8%)	
Sometimes	272 (49.9%)	69 (35.4%)	341 (46.1%)	
Many/all times	232 (42.6%)	117 (60.0%)	349 (47.2%)	
Breathing dust or gasses				<0.001
Never	135 (24.8%)	18 (9.2%)	153 (20.7%)	
Sometimes	283 (51.9%)	96 (49.2%)	379 (51.2%)	
Many/all times	127 (23.3%)	81 (41.5%)	208 (28.1%)	
Vibrating machinery				0.107
Never	154 (28.3%)	41 (21.0%)	195 (26.4%)	
Sometimes	282 (51.7%)	106 (54.4%)	388 (52.4%)	
Many/all times	109 (20.0%)	48 (24.6%)	157 (21.2%)	
Saws or sharp machinery				0.007
Never	108 (19.8%)	24 (12.3%)	132 (17.8%)	
Sometimes	253 (46.4%)	83 (42.6%)	336 (45.4%)	
Many/all times	184 (33.8%)	88 (45.1%)	272 (36.8%)	

#### Differences by reported serious injury status

We conducted univariate analyses to assess whether each of the work characteristics was associated with injury status. Results in Table 3 indicate that there were significant differences in all reported hazardous chemical exposures and working conditions by reported serious injury status, except in the case of the use of vibrating machinery (*p* > 0.10). Across the different analyses we consistently observed that a larger proportion of those who reported frequent hazardous exposure (many/all times) to each of the chemicals or hazardous working conditions also report a serious injury. Conversely, for uninjured LDLs, the proportion that reported “never” being exposed to hazards or conditions was greater when compared to injured LDLs.

#### Multivariable logistic regression analysis

The logistic regression analysis conducted to assess common predictors of serious reported injury over time revealed significant associations between hazardous chemical exposures and conditions and the likelihood of a reported serious injury in the integrated dataset. Exposure to gasoline and glue/adhesives, as well as working conditions that include the risk of getting cut, insufficient ventilation, and breathing dust or gasses, were significantly associated with reported serious injury (Table 4). The odds of a serious injury were significantly higher for workers exposed to gasoline “many/all times” (OR = 2.2, 95% CI: 1.15–4.26, *p* = 0.017) or “sometimes” (OR = 2.06, 95% CI: 1.37–3.09, *p* < 0.001) compared to those never exposed. Risk of being cut “many/all the time” (OR = 2.77, 95% CI: 1.51–5.28, *p* = 0.001) or “sometimes” (OR = 2.03, 95% CI: 1.14–3.77, *p* = 0.019) were significantly higher than for those never exposed to this risk. Insufficient ventilation increased the odds of injury (OR = 2.20, 95% CI: 1.25–3.90, *p* = 0.007) for participants reporting this exposure “many/all times” or “sometimes” (OR = 1.60. 95% CI: 1.01–2.57, *p* = 0.048), compared to those never exposed. Workers exposed to dust and gasses “many/all the time” (OR = 2.50, 95% CI: 1.34–4.82, *p* = 0.005) or “sometimes” (OR = 1.76, 95%CI: 0.99–3.22, *p* = 0.058) also had significantly higher odds of reporting a serious injury. On the other hand, workers frequently exposed to glue/adhesives “many/all times” had 64% lower odds of reporting a serious injury compared to those never exposed (95% CI: 0.16–0.78, *p* = 0.011).

**Table 4:** Final model results of multivariable logistic regressions in the integrated dataset.

Variable	Estimate	*SE*	Statistic	*p*-value	OR	95% CI	
Lead paint							
Never (ref)	–	–	–	–	–		
Sometimes	−0.237	0.216	−1.101	0.271	0.789	0.515–1.200	
Many/all times	0.266	0.345	0.772	0.440	1.305	0.659–2.561	
Solvents							
Never (ref)	–	–	–	–	–		
Sometimes	0.255	0.229	1.115	0.265	1.291	0.825–2.025	
Many/all times	0.311	0.304	1.023	0.306	1.365	0.748–2.470	
Glue or adhesives							
Never (ref)	–	–	–	–	–		
Sometimes	−0.302	0.222	−1.357	0.175	0.739	0.476–1.140	
Many/all times	−1.024	0.404	−2.537	0.011	0.359	0.159–0.778	
Gasoline							
Never (ref)	–	–	–	–	–		
Sometimes	0.722	0.207	3.493	0.000	2.059	1.374–3.093	
Many/all times	0.797	0.334	2.388	0.017	2.219	1.147–4.260	
Risk of getting cut							
Never (ref)	–	–	–	–	–		
Sometimes	0.708	0.303	2.338	0.019	2.031	1.143–3.773	
Many/all times	1.018	0.317	3.212	0.001	2.769	1.512–5.275	
Insufficient ventilation							
Never (ref)	–	–	–	–	–		
Sometimes	0.469	0.238	1.974	0.048	1.599	1.011–2.572	
Many/all times	0.788	0.290	2.720	0.007	2.198	1.249–3.895	
Breathing dust or gasses							
Never (ref)	–	–	–	–	–		
Sometimes	0.564	0.297	1.899	0.058	1.758	0.999–3.221	
Many/all times	0.915	0.326	2.808	0.005	2.498	1.337–4.823	

## Discussion

Our study explored the extent to which worker and work characteristics were associated with reported serious injuries among LDLs. We first tested whether there would be variability in these associations at different time points and then we tested for common predictors across time in a dataset that integrated four surveys (2014, 2019, 2020, and 2021). To our knowledge, our dataset on hazard and injury experiences of 740 LDLs in the U. S. is the largest of its kind and uniquely collected over a 7-year period. Its size and consistency in measurement and data collection procedures allowed us to test the proposed relationships. Below, we discuss the main results and their limitations.

### Reported injury

The proportion of LDLs in Houston, Texas, who reported experiencing a serious injury in the previous year is substantially higher than the 15% reported in a survey conducted in 2006 in Seattle, Washington (22), and the 19% that was reported in a national survey of day laborers conducted in 2004 (12). We observed variations by survey year in the proportion of study participants who reported a serious injury, ranging from 19.6% in 2014 to 39% in 2019. Furthermore, analyzing results over time in the integrated data set indicates that, overall, the proportion of workers who reported an injury is 27%. This is similar to the proportion (26%) reported by LDLs in San Francisco, California (24); but our definition of serious injury differed slightly. We included incidents in which the participant reported missing at least 1 day of work due to injury, went to work when injured but thought he should have stayed home, or had to receive medical attention from a doctor or clinic. By contrast, Burgel et al. (24) limited their definition of serious injury to one that leads to “missing work” but added “health complaint” as part of this measure. Comparability across studies would necessitate the adoption of a more standard definition and measurement of reported serious injury, as previously proposed (36).

### Worker characteristics

We assessed the extent to which demographic characteristics of LDL would predict reported serious injury. There were differences between the samples recruited in 2014, 2019, 2020 and 2021. The results indicate that there is significant variability in the demographic characteristics of LDLs recruited for the four surveys and trends that need to be explored further. For example, LDLs from Mexico have been the most prevalent over time, while the proportions of those from Cuba or El Salvador are the most variable. These changes in population composition confirm our own observations of how workers at the day labor corners have changed over time. However, none of the demographic characteristics, except for education in 2021, were significantly associated with a reported serious injury. Results were similar for the aggregated dataset, indicating that worker demographic characteristics do not increase the odds of reported serious injury. These results reinforce the necessity of addressing the characteristics of the work environment for injury risk reduction rather than the personal characteristics of the LDLs.

### Work characteristics

#### Specific hazardous chemical exposures and working conditions

We explored the influence of each hazardous chemical exposure and working condition and we found that all of the tested work-related hazards increased the odds of reporting a serious injury. These results were derived from univariate analyses, where each hazardous chemical exposure and working condition was examined individually as single predictors to assess its association with serious injury. However, when we explored their simultaneous influence in the multivariable logistic regressions (one for each survey dataset), only lead paint and gasoline were significantly associated with reported serious injury across all three examined surveys. In our final analysis using the integrated dataset, we tested for common predictors of reported injury and found that the results confirmed the influence of gasoline and lead paint, in addition to getting cut, insufficient ventilation and breathing gasses.

While it is clear that each hazardous chemical exposure and working condition (except glue/adhesives) significantly increased the odds of reported injury, it is not clear why their influence was attenuated and non-significant in the multivariable analysis. We tested for multi-collinearity using the Variance Inflation Factor and found no significant information overlap between the selected variables. Additional testing may be needed to disentangle the mutual influence among these work-related hazard exposures.

The hazardous exposures and working conditions identified in logistic regressions (gasoline, lead paint, getting cut, insufficient ventilation, and breathing gasses) are common in construction and landscaping jobs, which are the ones that LDLs are frequently hired to do (18, 21). For example, landscaping tasks involve using gasoline-powered mowers and trimmers, which generate dust and gasoline exhaust, as well as sharp tools and equipment for tree trimming.

Exposure to dust or gasses had a similar effect as a study of LDLs in Seattle in which “airborne hazards” were independently associated with increased odds of a reported serious injury. Dust and gasses are generated, for example, by operating power saws, sanding drywall, and cutting materials that contain silica.

We found that exposure to “gasoline” and “inadequate ventilation” (i.e., a potential indicator of hazardous chemical exposure) was associated with higher odds of a reported serious injury in one survey. Continuous exposure to hazardous chemicals such as gasoline and solvents adversely affects the central nervous system, causing headaches, dizziness, impaired cognition, and fainting, factors that can increase the risk of an injury (45, 46). On the other hand, exposure to glue or adhesives was significantly associated with decreased odds of serious injuries. In the construction sector, glues and adhesives are commonly used for tasks such as installing tiles, windows, doors, and flooring (47). These activities typically occur during the later stages of the construction process, when the use of heavy machinery and high-energy operations is minimal. As a result, the work environment during these phases tends to involve lower physical risk. The context in which there is frequent use of glues and adhesives (characterized by less hazardous tasks and more controlled conditions) likely contributes to our finding of a reduced likelihood of serious injuries.

Contrary to prior findings, the logistic regression results revealed unexpected insights of the working conditions associated with a reported injury. The literature indicates that falls to a lower level are a major cause of fatal and non-fatal injuries among workers in the construction industry (26, 28). Working conditions with the “risk of falling” among our participants, however, did not emerge as a factor related to a reported serious injury, despite LDLs’ frequently performing jobs in the construction industry. Similarly, overexertion injuries from lifting and lowering account for 30% of musculoskeletal disorders in the construction industry (34). “Lifting heavy weights,” however, was not a predictor of a reported serious injury among LDLs.

Our results also point to the complexity and variety of the jobs and tasks that LDLs frequently perform. It is possible that the risk of getting cut, having insufficient ventilation, and breathing dust or gasses may be experienced simultaneously, and the combinations of these and other conditions and exposures and their relationship to an injury among LDLs should be explored.

### Limitations

Our study has several limitations. First, we were able to explore the association between exposures and reported injury but were unable to determine the association with specific types of injuries. The nature of the injury related to each significant exposure remains a question to be addressed in a future study. Second, although we explored hazards relevant to LDL experiences, our scales were adapted from other studies, not developed locally and thus, there might be other important hazards that were not included in our surveys. Third, relying on cross-sectional surveys allows us to ascertain only the associations between exposures and serious injuries at discrete time points as well as comprehensively, but the nature of our data does not allow us to establish true temporality or the causal connections between exposures and serious injuries. Finally, we acknowledge that some changes in the significance level of exposures can be due to variation in sample sizes or simultaneous exposures in the multivariable model, and wide confidence intervals are mainly results of small sample size and high variance for estimations.

Our study has several strengths. First, by integrating data from four surveys, we had responses from 740 LDLs recruited, using the same random corner selection procedures at four points over 8 years. The large sample size provided stability to the reported results and increased the possible comparability to other groups of workers who experience similar working conditions. Second, we were able to analyze the relationship between reported serious injuries and a wide variety of potential exposures and working conditions relevant to the work experience of LDLs.

## Conclusion

Latino day laborers are a diverse and understudied population who work in a wide variety of jobs related mainly to the construction industry. They are frequently exposed to a wide variety of hazards and adverse working conditions, resulting in an increased risk of serious injuries. Our analysis provides valuable information about hazardous chemical exposures and working conditions encountered by LDLs that contribute to serious injury such as gasoline, lead paint, and risk of getting cut. Our findings can be used to develop, adapt and refine safety training for these workers that address specific hazards to better protect LDLs.

Our findings also provide lessons for practitioners and policymakers. When possible, injury prevention programs for Latino day laborers should include a rapid assessment of the jobs and/or tasks that they are hired to perform, as the risk for injury may vary with these demands. This initial assessment will help tailor safety interventions to the most relevant hazards currently experienced by LDL. While our results provide detailed information about hazardous chemical exposures and working conditions, future surveys may also focus on more explicit information about frequency of working on ladders, scaffolds, slippery surfaces or with saws, razor blades, or glass. Future research should also prioritize hazard-specific exposure studies that quantify the intensity, frequency, and duration of exposure with hazardous chemicals and working conditions. Additionally, research is needed to develop job task and injury risk profiles that identify which specific construction activities are most strongly associated with injury risk among LDLs.

These recommendations will improve the contextual relevance of safety training interventions and the practices that put workers at risk for injury. Similarly, while demographic characteristics helps to identify who is at risk, the emphasis of both data collection and intervention efforts should be on the exposure to hazards. While the demographic data provides a picture of the population, the exposure to hazards represent modifiable factors which Latino day laborers may have be able to control, reduce, or eliminate.

## Data Availability

The data that support the findings of this study are available from the corresponding author, Maria E. Fernandez-Esquer, upon reasonable request.
